# Immunohistochemical Expression of Myeloperoxidase in Placental Samples of Systematic Lupus Erythematosus Pregnancies

**Published:** 2016-06

**Authors:** Zahra Heidari, Hamidreza Mahmoudzadeh Sagheb, Nadia Sheibak

**Affiliations:** 1 Infectious Diseases and Tropical Medicine Research Center AND Department of Histology, School of Medicine, Zahedan University of Medical Sciences, Zahedan, Iran; 2 Department of Histology, School of Medicine, Zahedan University of Medical Sciences, Zahedan, Iran

**Keywords:** Lupus Erythematosus, Placenta, Trophoblasts, Peroxidase, Immunohistochemistry, Pregnancy Complications

## Abstract

**Objective:** Reports of increased level of Myeloperoxidase (MPO) in plasma and placental extracts of Systematic Lupus Erythematosus (SLE) has been proposed that MPO may have an important role in this pregnancy complication. In present study immunohistochemical MPO expression was investigated in placental samples of SLE women compared with normal controls.

**Materials and methods:** Ten patients with SLE were recruited as case group. Control group was selected from mothers with normal uncomplicated pregnancies. A monoclonal antibody specific for MPO was used for immunohistochemical staining and then the staining was quantified and differences between groups were compared using Mann-Whitney U test.

**Results:** There were significant differences in the expression levels of MPO in the syncytiotrophoblast cells and the extravillous trophoblast cells between the control and SLE groups (p < 0.05). There were no significant differences in the expression level of MPO in the vascular endothelium and the relative number of the MPO-positive leukocytes in placental tissue between SLE and control groups (p > 0.05).

**Conclusion:** The present study showed that MPO expression is increased in syncytiotrophoblast cells and the extravillous trophoblast cells of SLE placentas compared to healthy subjects. It seems that these changes are able to impress structure and function of placenta and survival of the fetus.

## Introduction

Systemic lupus erythematosus (SLE) is a chronic prototype, multisystem inflammatory and complex autoimmune disease that affects women in their childbearing years (1). This pervasive relapsing disease is characterized by diverse immune deposition, clinical manifestations and production of autoantibodies that lead to severe inflammation (1, 2).

Pathogenesis of SLE initiates with damage of vascular endothelial cells that active these cells and impresses other organs with angiophaty and vasculitis. SLE is a disease with unknown etiology that has high morbidity and mortality (2-4). Disease progression can be affected by many factors such as genetic background, change in metabolism of sexual hormones, high rate of apoptosis, increased oxidative stress levels and alerted cytokine profiles (2). 

Common long-term complications of SLE can lead to end-organ damage by affecting joints, skin, serosa, musculoskeletal, neuropsychiatric, renal, and cardiovascular systems due to the presence of autoantibodies to double stranded DNA (1, 5).

Because of the normal rates of fertility in SLE women, pregnancy is a usual occurrence. (5) There is an increased incidence of maternal and fetal morbidity and mortality in pregnant women with SLE. Pregnancy in these women is related to a higher risk of spontaneous abortion, premature birth, fetal growth restriction, placental abruption and preeclampsia. All of these complications are associated with placental dysfunction (6-8).

The placenta is a wonderful organ that interfaces between fetal and maternal components. It has an important function in the development and growth of the fetus. A major fetal component of the placenta is composed of placental chorionic villi. It is reported that any changes in placental constituents are probably able to endanger evolving fetus seriously (9).

About 13% of SLE patients suffer from preeclampsia (PE). Edema, thrombocytopenia, proteinuria and arterial hypertension are abnormalities that occur during both SLE and preeclampsia conditions. Both of these diseases may lead to fetal growth and vitality disorders. It was demonstrated that susceptibility to PE in SLE pregnant women with renal involvement was higher (4). Reports have suggested that PE and SLE probably have a common pathogenesis (5). Underlying pathology of both these, pregnancy complications have strong associations with vascular damage, increased oxidative stress and inflammatory responses (2, 10).

Myeloperoxidase (MPO), a marker of leukocyte recruitment, is associated with pathogenesis of some vascular disorders and inflammatory conditions (2, 11). MPO is a member of human peroxidase family that produced by neutrophils, macrophages and stimulated monocytes. This hemoprotein is a main source of reactive oxidants and is highly expressed in granules of polymorphonuclear cells (11-13). MPO functions in host defense at sites of inflammation and is involved in hormone synthesis and apoptosis inhibition (2, 14). Some studies reported that there was a strong association between human endothelial dysfunctions and serum levels of MPO (11). MPO has a dual role in the innate immunity, so that it can play as a microbicidal agent and be anti-inflammatory factor in some biological conditions (15).

Circulating MPO can be disassembled in the subendothelial spaces, leading to its aggregation in endothelial cell matrix (11, 16). The elevated levels of MPO presented in different inflammatory diseases have led to the hypothesis that MPO-mediated pathophysiological reactions have an important role in the progression of these diseases (13).

According to some reports, circulatory and placental extract MPO levels were increased in women with PE have shown significant increments compared with normal controls (11, 16). Immunohistochemical analysis confirmed the elevated levels of MPO in placental sections of preeclampsia patients (11). Significantly, increased plasma MPO concentrations have been also described in SLE patients    (12).

Telles et al. showed that there were higher plasma levels of MPO in SLE patients with arthritis in comparison with patients without arthritis (2). Therefore, it has been proposed that MPO may have a substantial role in PE and SLE pregnancy complications (10, 15, 16).

Based on our researches, until now there were not any data concerning MPO immunohistochemical expression in placental samples of SLE patients.

According to the above-mentioned evidences, we were promoted to examine expression of MPO in the placental sections of women with SLE. Thus, the aim of the present study was to compare the immunohistochemical expression of MPO in placenta of women with SLE and normal pregnancies.

## Materials and methods

This case-control study performed on total 20 placenta samples that were collected after delivery for paraffin embedding. 

Case group consist of 10 placentas from SLE patients that have antinuclear antibody level more than 10 and healthy placenta samples were considered as control group. Control subjects have no complications and underlying condition that affect normal pregnancy (Table 1).

The study was conducted in accordance with the Declaration of Helsinki and was approved by the Zahedan Medical University ethics Committee (IR.ZAUMS.REC.1394.13) for conducting research-involving humans. All subjects were enrolled after giving their informed consent to participate in the study.

Exclusion criteria included smoking, addiction and chronic diseases. Patients and controls characteristics and selection criteria are listed in our previous study (17).

**Table 1:** Clinical data of mothers in case and control groups

	**Patients** **n = 10**	**Controls** **n = 10**
Age of mother (year)	27 ± 7.80	25.20 ± 4.54
Placenta total volume(mm^3^)	395 ± 48.20	489 ± 97.26
Placenta weight (gr)	604 ± 33.11	550 ± 42.16
Umbilical cord diameter (mm)	8.33 ± 0.77	11.43 ± 0.84
Newborn weight (gr)	2775 ± 375.11	2775 ± 375.11

Values are given as mean ± SD

Placenta biopsies were fixed in modified Lillie’s solution (18), and cut into 5mm slices using systematic uniform random sampling (SURS). After routine tissue processing, samples were embedded in paraffin as described previously (17).

Immunohistochemistry (IHC) was used for localization of MPO within placentas from normal (n = 10) and SLE pregnancies (n = 10).

All IHC procedures were performed using the following staining protocol by Novolink polymer detection kit (RE7140-K, United Kingdom). All steps were done at room temperature (25°C) in a humidified chamber.

Tissue samples were cut to 4-µm thickness and were mounted on slides coated with a suitable tissue adhesive (Histogrip CL00-8050, Cedarlaine-Canada). Sections were allowed to air dry at room temperature overnight. Then the sections were deparaffinized, rehydrated and finally were washed in distilled water.

All sections for immunostaining were processed for heat-induced antigen retrieval. Slide-mounted sections immersed in 0.01 M sodium citrate buffer (pH 6.0) were placed in a laboratory autoclave (1 cycle, 120˚C for 10 min).

To neutralize endogenous peroxidase activity, sections were blocked with a peroxidase-blocking reagent (Novacastra peroxidase-United kingdom), for 10 min and were washed in a bath of Tris buffer (0.05 M Tris-HCl, pH 7.0–7.6).

Then, the slides were incubated with Protein-Block (Novacastra-United Kingdom) for 5 minutes at room temperature, followed by overnight incubation with the anti-MPO primary antibody (mouse monoclonal, Novacastra-United kingdom) at 4°C. Sections were incubated with Novolink Post Primary for 30 min and then with Novolink Polymer for 30 min at room temperature.

Peroxidase activity was developed with a DAB working solution (100µl of DAB Chromogen added to 1ml of Novolink DAB Substrate Buffer) for 30 minutes.

Sections were counterstained with Mayer’s hematoxylin for 5 min and were rinsed in fresh distilled water. Sections were dehydrated and mounted with Entellan (Merck).

Positive controls (colon carcinoma) and negative controls (omission of primary antibody) were run for each batch of slides. Two examiners detected immunoreactivity microscopically on masked label slides.

Evaluation of MPO staining was based on a qualitative scale of -, +, ++, +++ representing no, weak, moderate or strong staining as described by Salem et al (19).

Conversion of the qualitative data to ordinal data (0-3) was used for the MPO IHC analyses in order to permit statistical analysis.

Estimation of the relative number of leukocytes performed using by a square-shape grid. For each section, four fields (X400 magnification) were selected randomly under a photomicroscope by movement of stage in X and Y directions and number of MPO positive stained leukocytes were counted.

The Statistical Package for Social Sciences (SPSS) 16.0 software was used for statistical calculations. Values were presented as mean ± SEM, and differences were compared by using Mann-Whitney U test. In all evaluations, p values less than 0.05 were regarded as significant.

## Results

**Figure 1:**
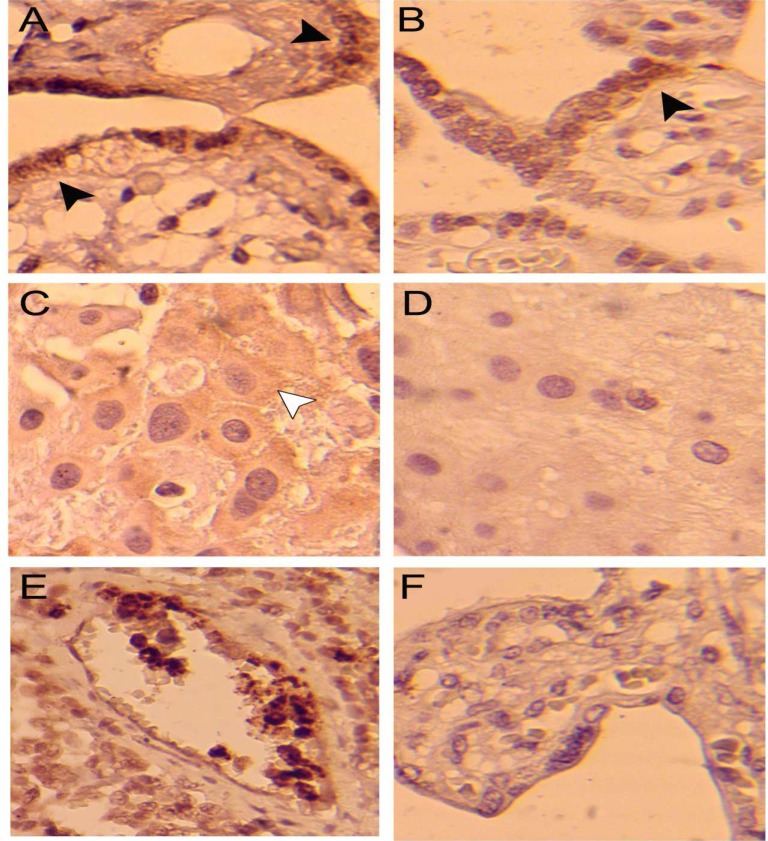
Immunohistochemical staining of myeloperoxidase in SLE placenta (A and C), healthy placenta (B and D) and Colon carcinoma as positive control (E) and SLE placenta without primary antibody incubation used as negative control (F). Myeloperoxidase positive expression in synchiotrophoblast (black arrowheads) and extravillous trophoblast of placenta (white arrowhead) are shown

Results of the immunohistochemical staining of the placental sections in both groups have been shown in figure 1 and table 2.

The expression levels of MPO in the syncytiotrophoblast cells had statistically significant differences between the control and SLE groups (p < 0.05). There were also statistically significant differences in the expression level of MPO in the extravillous trophoblastic cells of the placenta between SLE and healthy subjects (p < 0.05). There were no statistically significant differences in the expression level of MPO in the placental vascular endothelium between SLE and control group (p > 0.05).

The relative number of MPO-positive leukocytes in the lupus group were increased compared to the control group, but this difference was not statistically significant (p > 0.05). 

**Table 2:** Results of immunohistochemical expression of myeloperoxidase in placenta samples

MPO expression in placenta	Patients n = 10	Controls n = 10	p value
**Vessels endothelium**	**0.20 ± 0.200**	**0**	**0.662**
**Syncytiotrophoblast**	**2.00 ± 0.447**	**0.50 ± 0.224**	**0.020** a
**Extravillous trophoblast**	**1.60 ± 0.400**	**0.50 ± 0.224**	**0.035** a
**MPO + leukocytes**	**4.20 ± 0.916**	**2.00 ± 0.365**	**0.090**

Values are given as mean ± SEM;

a Statistically significant result at p value < 0.05

## Discussion

The present study showed that MPO highly expressed in syncytiotrophoblast cells and the extravillous trophoblastic cells of SLE placenta compared to healthy subjects. Until now, we could not find similar study on immunohistochemical expression of MPO in placental sections of SLE patients.

Pathogenesis of SLE is related to inflammation during the severe stages of disease. It is indicated that increased oxidative stress may be responsible for SLE progression (15). Since, MPO is a marker for identification of activated neutrophils and monocytes; it can be used as a marker for oxidative stress elevation (16).

However, Association of elevated oxidative stress with immune dysregulation during SLE and its molecular basis remain unknown. It is identified that oxidative stress lead to syncytiotrophoblast dysfunction, which cause release of some inflammatory factors (20). 

Zhang et al. localized MPO by IHC in placentas of normal pregnancy. They showed that this human peroxidase was expressed in the cytoplasm and nucleus of syncytiotrophoblastic cells (14). This is similar to our findings in the control group. The MPO expression in control samples is probably a response to the stress induction that occurs during and after delivery (14). 

Gandley et al.(11) demonstrated that rates of MPO-positive syncytiotrophoblasts in placental villi from PE patients was greater than healthy samples (11). Our results proposed the increased expression of MPO in syncytiotrophoblast cells during SLE. It could be concluded that syncytiotrophoblast dysfunction probably is able to induce additional oxidative stress to maternal tissues during implantation and may lead to lupus progression to PE.

Our finding showed that MPO expression in extravillous trophoblastic cells of basal plate increased significantly compared to control group. 

Gandley et al. found increased expression of MPO in cytotrophoblasts of basal plate from PE women compared to normal pregnancy (11). They reported that positive cells for MPO staining in placenta samples were infiltrating white blood cells, syncytiotrophoblast and trophoblast cells that confirmed by our findings.

Trophoblastic cells invaded to the maternal spiral arteries during implantation (about 8^th^ weeks of pregnancy). These fetal-derived cells include two population, cytotrophoblast and syncytiotrophoblasts that involved in placental villi structure. Syncytiotrophoblasts be composed of the outer trophoblastic layer that is interrelated with the mother endometrium to fetal-maternal contact and attachment. Uncommon immune markers including immune suppressor and immune regulator molecules could be expressed by placental trophoblasts. Immune-related function of trophoblast is involved in the improvement of fetal tolerance and pregnancy outcome (21).

Therefore, increased expression of MPO in the both trophoblastic cell populations may be caused by systemic immune reactions that occur during SLE.

The results of the present study showed that immunohistochemical expression of MPO in leukocytes population was high and relative number of these cells increased in SLE samples, but these changes were not statistically significant compared to healthy controls.

Systemic inflammation and T-cell activity during lupus may result in development of pregnancy complications in the SLE patients (5).

Aly et al. showed increased generation of superoxide by maternal activated neutrophils during PE in comparison with normal pregnancies that probably lead to endothelial disruption induced by syncytiotrophoblast microvillous membrane (STBM) (22). As, the STBMs are in direct exposure with maternal blood circulation and especially maternal neutrophils, interaction between maternal leukocytes and STBMs probably could exacerbate endothelial disorder in PE by released cytokines and oxygen free radicals (22). Neutrophils act against bacteria by releasing the contents of their granules and have important roles in nonspecific immune reactions (23). It is demonstrated that, aggregation of MPO on the neutrophils surfaces during pregnancy can lead to production of elevated rates of oxidants by these cells (16).

Our finding showed that there is no significant difference in MPO expression level in the placental vascular endothelium of SLE patients in comparison with control group. One reason for this finding is probably that placental vascular endothelium has not direct contact with maternal blood as a MPO source (21). In addition, histological structure, oxidative stress level and oxygen access may differ in various placental regions (24). It has been shown that all placentas are not similarly affected (7). MPO level in the central portion of normal placenta was higher than its peripheral parts (23). As we used SURS for the placentas sampling, all parts of each placenta had the equal chance for selection and the results are more reliable than previous studies (17).

Fetal tolerance to maternal immune response performed through protective functions of placenta, which inhibit immunological confliction. Immunological interaction between fetus and maternal immune system considerably could be able to affect pregnancy outcome (23). 

Prokopenko et al. indicated that there was a close association between the possible pregnancy outcome and the placental MPO level (23). In other hand, Salamonsen et al. indicated that endometrial leukocytes might have important roles in establishment and altered quantity of the gestation(25). 

Delivery mode is another effective factor in the cellular response of placenta and impresses oxidative stress rates in women around parturition time. For example, Plasma concentration of MPO showed an elevation in patients after cesarean section and during vaginal delivery that probably was due to stress induction by surgery or labor respectively (16). 

Reports noted that there was a significant difference in the MPO level of plasma and placental extracts between PE samples and healthy group (11, 16). Increased circulatory level of MPO in women with PE might cause by exacerbated immune responses (16). The susceptibility to preeclampsia in pregnant woman with SLE probably is higher. PE in lupus patients may be triggered by inflammatory response. Reports suggested that immune dysfunction in SLE may put pregnancies at high risk for development of PE (5).

Our findings proposed that an elevated MPO expression level in placenta tissue probably could be a key factor that triggers PE in pregnant SLE patients.

Finally, we concluded that the immunohistochemical expression levels of MPO in the syncytiotrophoblast cells and the extravillous trophoblast cells had statistically significant differences between the control and SLE groups. These changes may explain the SLE pregnancy problems. Further studies are required to elucidate the role of MPO in the regulation of placental development and function.
